# Bio-Inspired Radio-Frequency Source Localization Based on Cochlear Cross-Correlograms

**DOI:** 10.3389/fnins.2021.623316

**Published:** 2021-02-16

**Authors:** Yingying Wang, Soumyajit Mandal

**Affiliations:** ^1^Department of Electrical, Computer, and Systems Engineering, Case Western Reserve University, Cleveland, OH, United States; ^2^Department of Electrical and Computer Engineering, University of Florida, Gainesville, FL, United States

**Keywords:** RF cochlea, spectrum analysis, source localization, cognitive radio, cross-correlograms

## Abstract

This paper describes a bio-inspired radio frequency (RF) scene analysis system based on cross-correlating the outputs of two single-chip RF spectrum analyzers. The latter are implemented using digitally-programmable “RF cochlea” chips (in 65 nm CMOS) that integrate a transmission-line active cochlear model, consisting of 50 parallel exponentially-spaced stages for analyzing the radio spectrum from 1.0 to 8.3 GHz, together with an output encoding network. The encoders convert the analog outputs of all cochlear stages into parallel delta-sigma (Δ-Σ) modulated digital signals for real-time demodulation and analysis by a digital back-end processor. These outputs can also be multiplied with each other to generate cochlear correlation matrices (known as cross-correlograms). Simulation results demonstrate the use of cross-correlograms for wide-range time-delay estimation and real-time multi-source localization at different frequencies and input signal-to-noise (SNR) ratios. Over-the-air measurement results from an experimental two-channel RF scene analysis prototype confirm the use of such time-delay estimates, which are analogous to interaural time differences (ITDs) in the auditory system, for azimuthal source localization at 3.4 GHz. In addition, differences in received signal strength at the two cochleas, which are analogous to interaural level differences (ILD) in biology, are also used to localize RF sources.

## 1. Introduction

The mammalian auditory system has the ability to detect, analyze, and segregate multiple sound sources in noisy environments (Geisler, 1998). This process starts when vibrations of the tympanic membrane caused by incident sound waves are transduced into motion of the basilar membrane (BM) within the inner ear (cochlea). The cochlea contains a sophisticated signal processing system that converts BM motion into a time-varying pattern of neural excitation on the auditory nerve while consuming only ~14 μW of power (Sarpeshkar, 2010). Cochlear outputs are further processed by higher centers in the auditory nervous system to generate the perception of sound, resulting in exquisite sensitivity and over 120 dB of input-referred dynamic range (Pickles, 2013).

**Figure 1:**
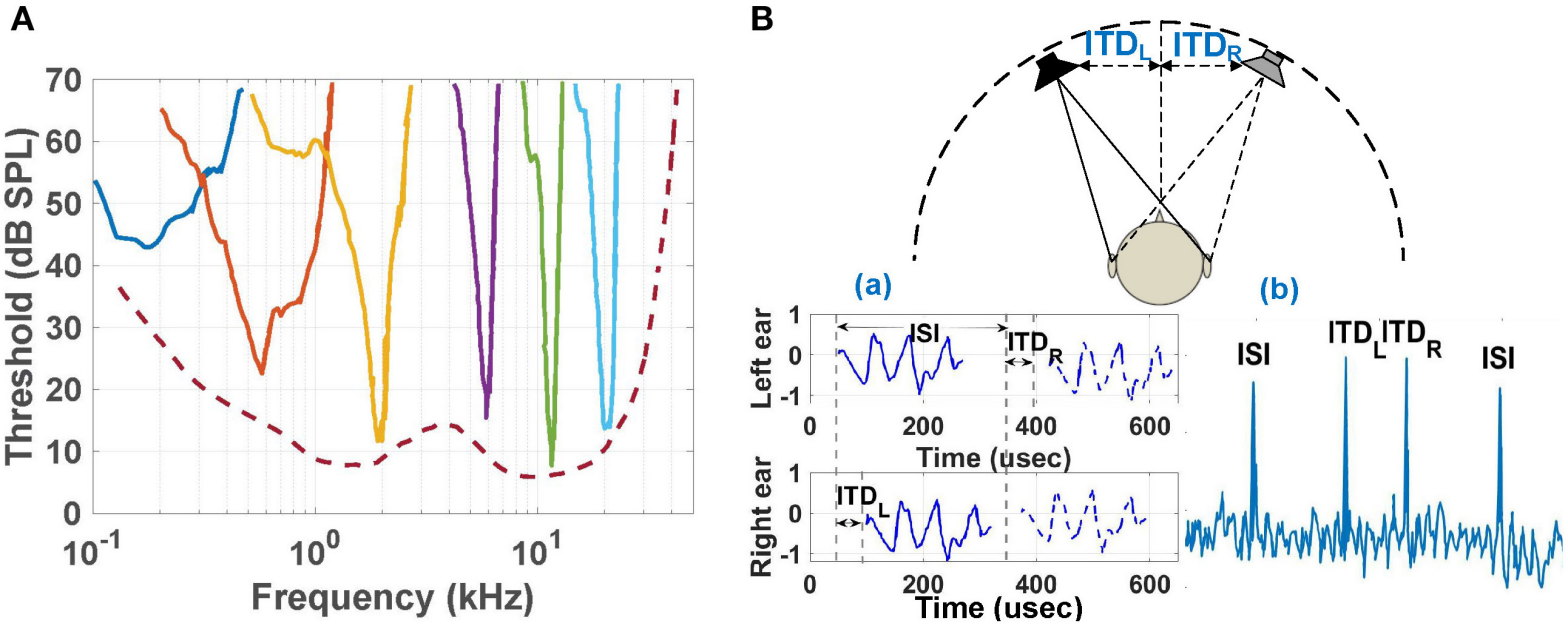
**(A)** Neural frequency-threshold tuning curves in monkey (macaque) for 9 selected frequencies, adapted from Joris et al. (2011) and Verschooten (2013). **(B)** (a): Schematic of the input signals to the two ears when two loudspeakers, separated in azimuth, emit identical sounds with a slight delay (*ISI*). *ITD*_*L*_ and *ITD*_*R*_ signify the interaural time differences between signals from the two source locations for the left and right ears, respectively; (b): Cross-correlation between the two ears for broad-band outputs from the speakers. Four peaks occur at correlation delays (τ) corresponding to ±*ISI*, *ITD*_*L*_, and *ITD*_*R*_. Figure adapted from Keller and Takahashi (1996).

Frequency selectivity and neural phase-locking are two fundamental properties of the peripheral auditory nervous system. These properties originate in the cochlea and are pervasive in behavioral and neural responses (Verschooten, 2013). Frequency selectivity refers to the ability to resolve individual spectral components of complex sounds and is important for the accurate perception of, for example, speech. Auditory nerve (AN) fibers that innervate inner hair cells (IHCs) along the BM are most sensitive to a particular “characteristic” tonal frequency. The tuning of AN fibers is classically described by a frequency-threshold tuning curve (FTC; as shown in Figure 1A). On the other hand, neural phase-locking is the property of AN fibers to lock to the instantaneous pressure fluctuations in the waveform of sounds. Phase-locking is essential for binaural hearing, i.e., spatial localization of sound sources in azimuth (left-right), and is also thought to be invoked in certain aspects of perception, such as pitch, loudness, and speech perception (Carney, 1994; Moore, 2008).

Spatial localization of sound sources is one of the key aspects of auditory perception (Popper et al., 2005). The auditory system of mammals and birds relies on a combination of monoaural and binaural cues to localize sources, of which the most important binaural cues are interaural time difference (ITD) and interaural level difference (ILD). Here ITD is the difference in arrival time of a sound between two ears, while ILD is the (frequency-dependent) difference in its intensity (i.e., loudness). The output of a cross correlation-like computation based on the ITDs (as shown in Figure 1B) is displayed as neural activity across the auditory space map in the cochlear nucleus (CN). These peaks are further processed by higher stages of the auditory pathway to associate source locations with other properties (frequency, intensity, temporal structure, etc.), thus generating an *auditory scene map* (Bregman, 1994).

Given that acoustic and electromagnetic wave propagation involves similar physics (propagation, absorption, and scattering), it is interesting to consider whether similar scene analysis principles may be useful for analyzing radio frequency (RF) environments. In fact, auditory source localization concepts have clear analogs for RF signals. For example, we recognize that ITD can be used to estimate the angle of arrival (AOA) (as in traditional beamformers), while the ILD is a type of differential received signal strength indicator (RSSI) measurement (Mead et al., 1991; Chan et al., 2007) that may also be useful for AOA estimation. Thus, hardware-efficient ITD and ILD estimators for RF signals would enable energy-efficient RF source localization, which in turn would be of significant interest for a variety of spatial processing tasks in wireless systems, including (i) beam management for MIMO transceivers (Xue et al., 2018), (ii) dynamic spectrum access (DSA) algorithms for cognitive radio (CR) networks (Dhope et al., 2013), and (iii) interference/clutter rejection in radar processors (Chen and Vaidyanathan, 2008; Gu et al., 2018). The resulting location estimates can be combined with other source properties (frequencies, modulation types) to generate so-called “RF scene maps”. Such maps can be used, for example, by beam management and DSA algorithms within a CR network to minimize multi-user interference and maximize user-perceived data throughput.

A wide variety of algorithms have been used for 1D, 2D, or 3D localization of both electromagnetic and acoustic sources, including delay-and-sum (DAS) or discrete Fourier transform (DFT)-based linear beamforming, multiple signal classification (MUSIC) (Schmidt, 1986) and its variants (Zhang and Ng, 2009), and generalized cross-correlation (GCC) (Knapp and Carter, 1976; Balestrieri et al., 2020). Most of these algorithms require matrix operations (multiplication, inversion, or eigen decomposition) that need to be implemented digitally^1^. As a result, a complete RF receiver chain and high-speed high-resolution ADC is required per sensor (i.e., antenna element), which becomes energy-inefficient for ultra-broadband sensing and/or large-scale arrays. Hybrid analog-digital beamforming has been proposed as a hardware-efficient alternative to this problem (Sohrabi and Yu, 2016). However, while the resulting linear beamformers are suitable for separating source data streams (e.g., for MIMO), they are inferior to non-linear algorithms such as MUSIC or GCC in terms of source localization accuracy.

Here, we consider a hardware-efficient hybrid analog-digital approach to the problem of simultaneously localizing multiple narrowband RF sources over an ultra-broadband frequency range using very few (ideally only two) sensors. The system uses an ultra-broadband “RF cochlea” chip developed in our prior work (Mandal et al., 2009; Wang et al., 2020). This chip contains a cochlear pre-filter that extracts a set of lower-bandwidth RF features from wideband inputs (including amplitude, phase, and frequency information for spectral occupancy and modulation recognition) while preserving both ITD and ILD information. Features from multiple spatially-separated RF cochleas are then compared to efficiently compute source locations. In particular, we find ITDs by computing an outer product of the outputs of two cochleas, which is known as the *stereausis algorithm* (Shamma et al., 1989). In particular, the stereausis algorithm computes interaural differences by combining ipsilateral inputs at a given characteristic frequency (CF) with contralateral inputs from locally-off-CF locations. These operations are hardware-efficient, and experiments with audio sources suggest that it results in comparable localization accuracy as MUSIC and other well-known algorithms (Pham et al., 1999; Julian et al., 2004).

This paper is organized as follows. In section 2, we describe the system-level design of the cochlea-based RF source localization system and the simulation results. Measurement results from an experimental two-channel prototype are presented in section 3. Finally, section 4 concludes the paper.

## 2. System Design and Simulations

### 2.1. Theoretical Background

**Figure 2:**
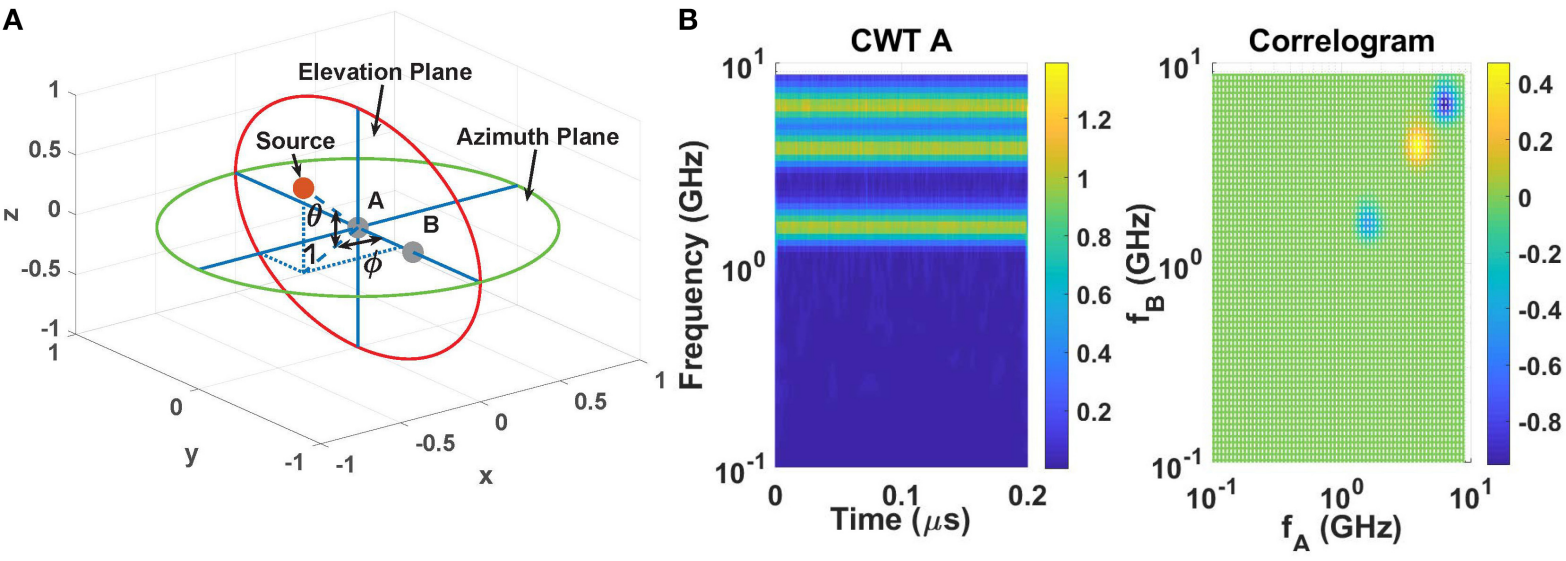
**(A)** Geometry for 2D far-field source localization. **(B)** Continuous wavelet transform (CWT) of signal at sensor A (tones at 6.3, 3.9, and 1.6 GHz) (left), and the cross-correlogram (right) generated by correlating it with the CWT of signal at sensor B (same frequencies, but with phase shifts of π, −π/3, and 2π/3 rad, respectively).

The far-field 2D source localization problem (as shown in Figure 2A) consists of estimating the location of signal source(s) in both the azimuth plane (angle ϕ) and the elevation plane (angle θ) using signals received by an array of *N* sensors (the *N* = 2 case is shown in the figure). We simplify this general problem by making two simplifications. Firstly, we ignore elevation and only focus on 1D localization in the azimuth plane using *N* = 2 antennas (as shown in Figure 2A). Secondly, we assume that the sources are non-overlapping in the frequency domain. Given the wave velocity in the medium, source locations can now be found by estimating the time delays between signals received by the sensor array.

Given sources with non-overlapping spectra, it is natural to consider architectures that decompose the incoming broadband RF waveforms from the antennas into non-overlapping frequency bins (“channels”) and run independent single-source localization algorithms on each channel. However, these operations should be performed in the analog domain to eliminate the need for (i) high-speed digitization of the entire broad RF bandwidth (which can exceed several GHz for high-end CR applications), and (ii) power-hungry digital signal processing. In this case, continuous-time analog filter-banks are a natural choice for performing the necessary frequency decomposition. We use fully-integrated single-chip models of the mammalian inner ear (cochlea) for this purpose, since they behave as hardware- and power-efficient filter-banks at both audio and radio frequencies (Sarpeshkar et al., 1998; Mandal et al., 2009; Wang et al., 2020). These chips mimic the exponentially-tapered structure of the fluid-filled biological cochlea using either bidirectional transmission lines or filter cascades.

The outputs of our cochlear models (in the linear regime) are well-modeled as constant-*Q* frequency bins, which in turn resemble those generated by a continuous wavelet transform (CWT) (Yao and Zhang, 2002). To illustrate the expected frequency decomposition results, Figure 2B (left panel) shows the CWT for a three-tone input at 6.3, 3.9, and 1.6 GHz that is fed into antenna A, In addition, a time-delayed input (with individual phase shifts of π, −π/3, and 2π/3 rad, respectively) is fed into antenna B. These time-delays can be estimated by multiplying and low-pass filtering the two sets of *N*-element CWT outputs at each time step, resulting a (possibly time-varying) *N* × *N* 2D matrix we refer to as a *cross-correlogram*. Denoting the two complex CWT output vectors as **w_*A*_** and **w_B_**, each element in the cross-correlogram is given by

(1) $$\begin{array}{r} x_{AB,i,j}\left(t\right)=|w_{A,i}\left(t\right)||w_{B,j}\left(t\right)|\cos\left(\Delta \psi_{AB,i}\right), \end{array}$$

where ψ_*A*_ and ψ_*B*_ are the phases of **w_*A*_** and **w_B_**, respectively, and Δψ_*AB, i*_≡(ψ_*A,i*_−ψ_*B,i*_). Note that this wavelet-based cross-correlogram algorithm is simply a scale-localized version of the usual inner product between two signals.

The time-averaged cross-correlogram **X_AB,av_** for the three-tone input signals considered earlier is shown in Figure 2B (right panel). The good frequency resolution of the CWT results in localized structures along the main diagonal (*i* = *j*) at specific scales corresponding to the input frequencies ω_*i*_, and their normalized amplitudes *x*_*n,i,j*_ = *x*_*AB,i,j*_/(|*w*_*A,i*_(*t*)||*w*_*B,j*_(*t*)|) encode the time delay τ_*i*_ = (Δψ_*AB,i*_)/ω_*i*_ for each component. Specifically, the time delay is estimated as

(2) $$\begin{array}{r} \tau_{i}=\frac{\cos^{-1}\left(x_{n,i,i}\right)}{\omega_{i}}. \end{array}$$

The ambiguity-free range for this type of time delay estimation is [0, π/ω_*i*_], i.e., half an RF cycle, with maximum sensitivity *d*(*x*_*n, i, i*_)/*d*(Δψ_*AB,i*_) = 1 around the zero-crossing (Δψ_*AB,i*_ = π/2). It is interesting to compare this result with a linear two-element DAS or DFT beamformer, for which the output amplitudes of the two beams are proportional to [cos(Δψ_*AB,i*_/2)±sin(Δψ_*AB,i*_/2)]. By contrast, Equation (1) shows that the cross-correlogram is proportional to cos(Δψ_*AB,i*_). Clearly, cross-correlograms provide ~2 × higher resolution for estimating small time delays than linear beamformers. However, they also remove all common-mode phase information (e.g., due to data modulation) present in the two inputs, so data cannot be recovered from the cross-correlogram (unlike for linear beamformers).

Finally, note that the off-diagonal elements in **X_AB,av_** are almost zero because of the orthogonality of the CWT outputs. The benefits of including these off-diagonal terms for delay estimation will be explained later.

### 2.2. System Architecture

**Figure 3:**
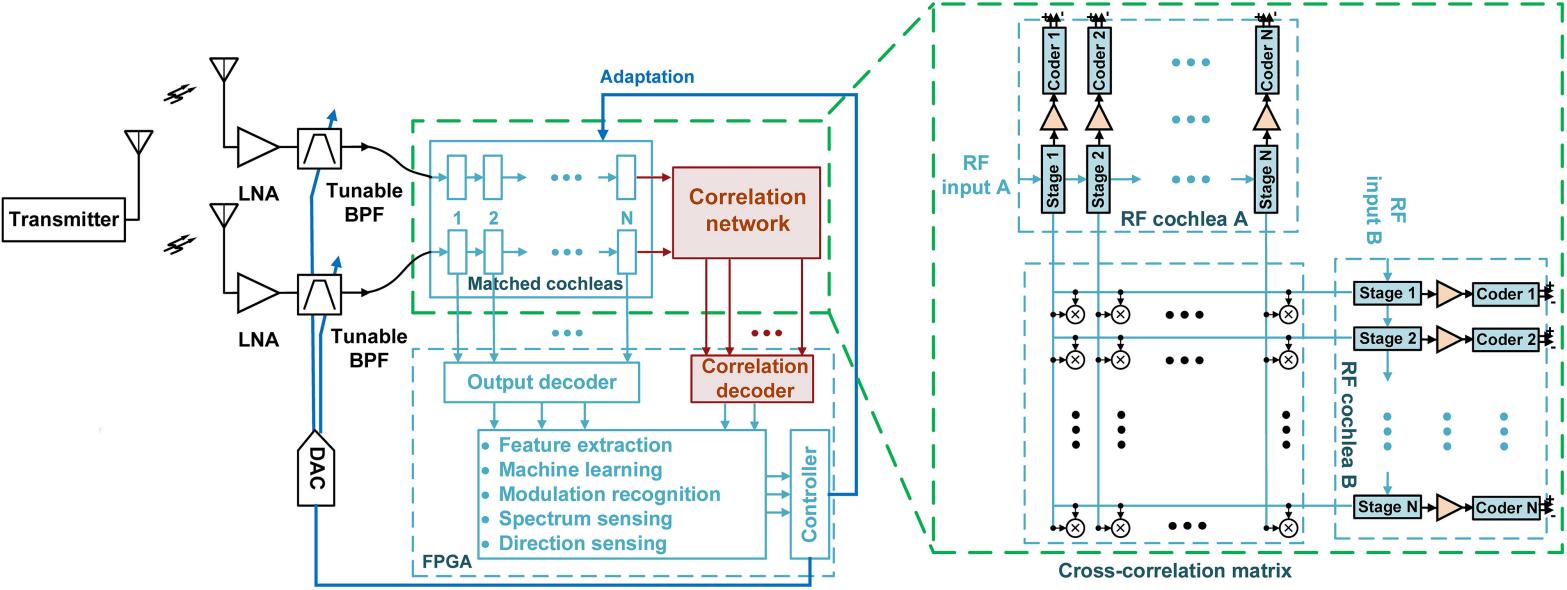
Detailed block diagram of the proposed adaptive radio frequency (RF) scene analysis system. This paper focuses on the highlighted blocks (the correlation network and correlation decoder).

A block diagram of the proposed cochlea-based broadband RF scene analyzer is shown in Figure 3. Two spatially-separated broadband antennas sense the local EM field. Each antenna output is amplified and filtered [resulting in the RF transfer function *H*_*RF*_(ω)], and then decomposed into a set of *N* CWT-like frequency bins by an RF cochlea. The signals in these bins are denoted by the vectors **w_*A*_**(*t*) and **w_B_**(*t*), respectively. Low-complexity on-chip circuits then extract a set of RF features (including signal amplitude, phase, and frequency) from each bin. To simplify later processing, **w_A,B_**(*t*) is encoded using polar coordinates, such that each complex element *Ae*^*jψ*^ is represented using separate amplitude (*A*) and phase (ψ) components. The chips digitally encode both these components on single wires: amplitudes using integrated 1-bit Δ-Σ modulators, and phases using the phase-locked outputs of integrated injection-locked frequency dividers (ILFDs). By enabling such “slow-and-parallel” digitization of lower-bandwidth features, the RF cochleas significantly improve system-level energy efficiency compared to conventional real-time signal analyzers that directly digitize the broadband RF waveform (Wang et al., 2020). Finally, the digitized CWT-like output vectors **w_A,B_**(*t*) of each cochlea are analyzed by a digital processor (e.g., the FGPA shown in Figure 3) to extract relevant features of the scene, including source frequencies and power levels.

In addition to independently analyzing each cochlea's output, Figure 3 shows that the RF scene analyzer also uses a 2D network of multipliers (known as the stereausis network) to calculate the outer product of the vectors **w_A,B_**(*t*) generated by the two cochleas, thus generating the *N* × *N* cochlear cross-correlogram matrix **C_AB_**(*t*). We will show that in many cases it is sufficient to generate an approximation of **C_AB_**(*t*) by cross-correlating only the relative phases of **w_A,B_**(*t*) (i.e., by using only the phase-locked ILFD outputs). In this case, the multipliers in the stereausis network can be replaced by XOR gates, which act as 1-bit multipliers and thus greatly simplify the circuit implementation. The digital processor can now extract features from the cross-correlogram matrix **C_AB_**(*t*) to estimate relative time delays and source locations, which is the focus of this paper.

To complete the process of scene analysis, the two sets of extracted features (from the individual cochleas and the cross-correlogram, respectively) are fed into machine learning (ML) algorithms, such as the deep belief networks (DBNs) that were used for modulation recognition in our previous work (Wang et al., 2020). Finally, the outputs of these algorithms can be used to optimize the RF transfer function *H*_*RF*_(ω), e.g., by tuning the center frequency and/or *Q* of a band-pass filter (BPF) as shown in Figure 3. Such ML-driven closed-loop adaptation can significantly improve the system's dynamic range (DR) by either emphasizing desired signals or canceling unwanted signals (i.e., blockers) (Wang and Mandal, 2017).

### 2.3. Architecture of the RF Cochlea Chip

**Figure 4:**
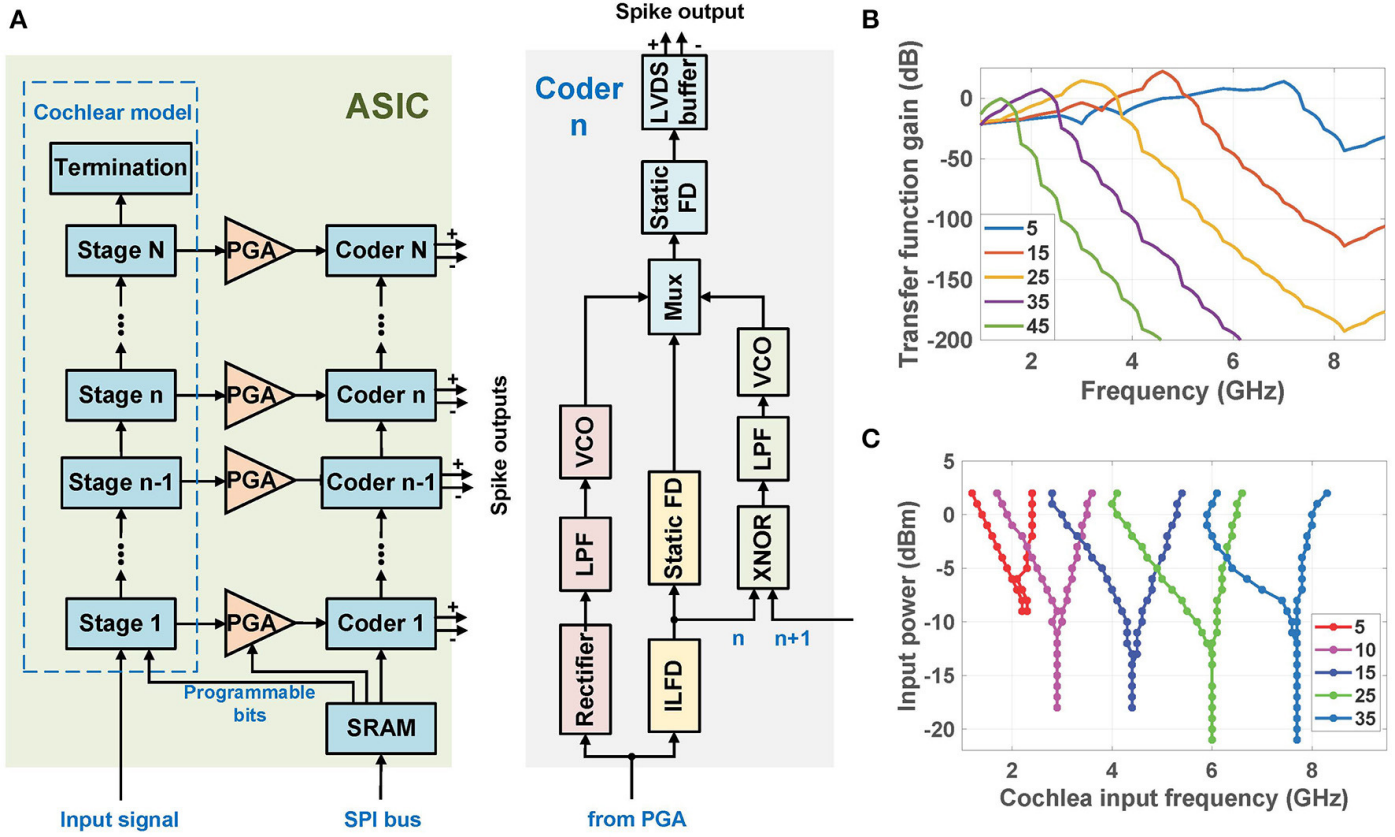
**(A)** Simplified block diagram of the cochlea-based ultra-broadband RF spectrum analyzer chip (Wang et al., 2020). **(B)** Simulated small-signal spatial transfer functions of the amplitude encoders to continuous-wave (CW) inputs at stages {5, 15, 25, 35, 45} (Wang et al., 2020). **(C)** Simulated input sensitivity curves of the frequency and phase encoders (divide-by-3 ILFDs) at stages {5, 10, 15, 25, 35}.

Each digitally-programmable RF cochlea chip (Wang et al., 2020) includes a transmission-line active cochlear model with 50 exponentially-spaced stages that analyzes the radio spectrum from 1.0 to 8.3 GHz. Each output is processed by three encoder circuits that are sensitive to signal amplitude, frequency, and phase delay between adjacent stages, respectively (as shown in Figure 4A). The amplitude components of all stages are digitized in parallel using on-chip Δ-Σ modulators, as mentioned earlier. Figure 4B shows the simulated small-signal transfer functions of the amplitude encoders in several stages to continuous-wave (CW) inputs from 1.0 to 9.0 GHz. These functions have asymmetric band-pass shapes with cutoff locations that move logarithmically toward later stages as the frequency decreases, resulting in a CWT-like filter bank but with some overlap between the channels. In particular, the amplitude *V*_*out*_(*n*) of the *n*-th cochlear stage reaches its maximum at a characteristic or “best” frequency

(3) $$\begin{array}{r} \omega_{c}\left(n\right)=\omega_{c}\left(0\right)\exp\left(-n/N_{nat}\right), \end{array}$$

where *N*_*nat*_ = 24 is a design constant.

Each frequency encoder uses a ring-oscillator-based divide-by-3 ILFD whose locking range depends on both its free-running frequency and the amplitude of the injected signal. In particular, the ILFD in the *n*-th stage locks to signal components within *V*_*out*_(*n*) that are relatively close to its free-running frequency, which is designed to approximately match ω_*c*_(*n*). The simulated locking sensitivity curves of the ILFDs in several stages are shown in Figure 4C. Each circuit locks when the input power level at any particular frequency exceeds the plotted value at that point, and its phase in the locked state tracks that of the injected signal. Thus, the ILFD outputs encode both signal frequency and phase. These phase-locking curves are qualitatively similar to those observed in the mammalian cochlea (see Figure 1A).

### 2.4. Delay Estimation Using Cochlear Cross-Correlograms

**Figure 5:**
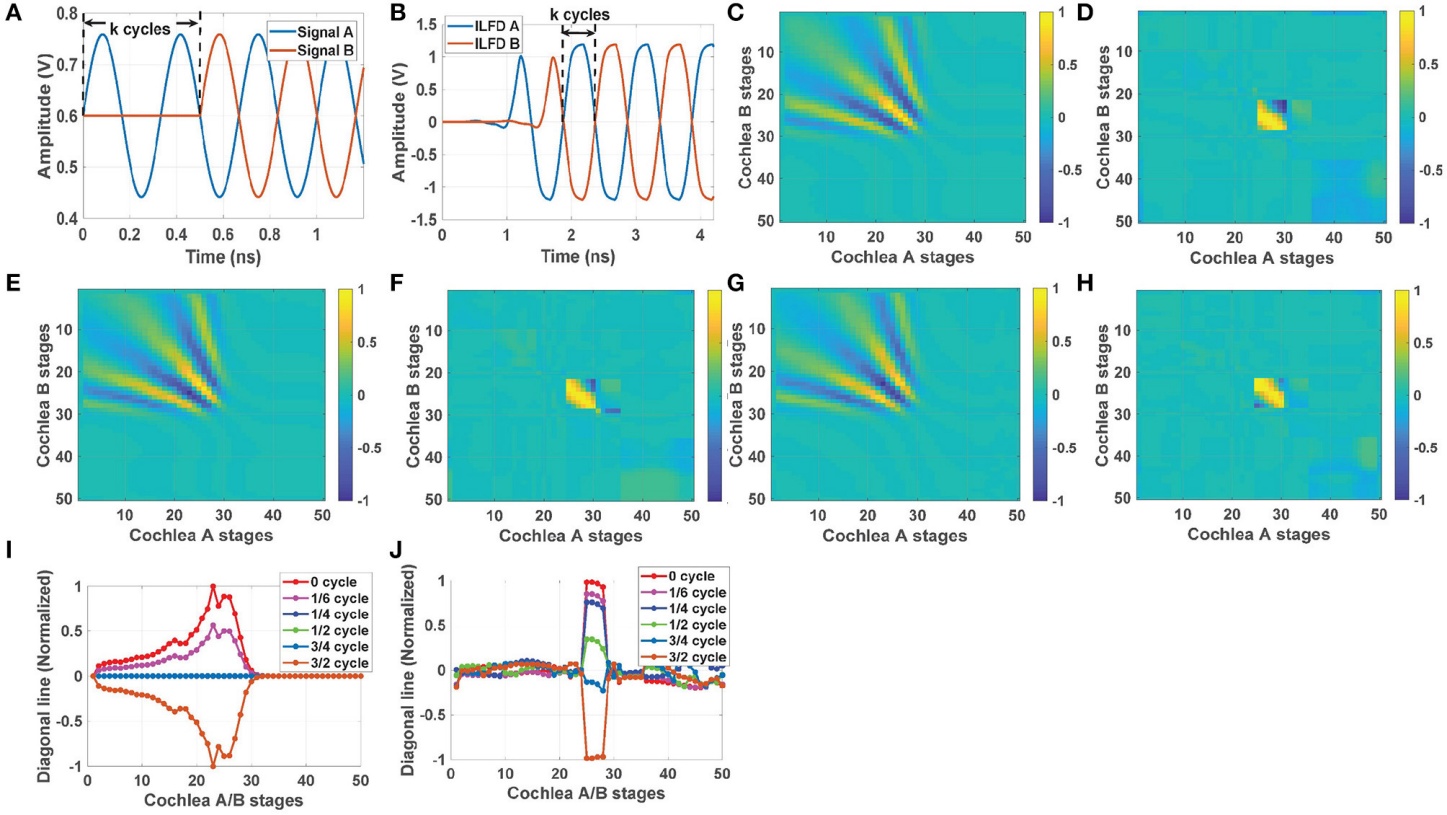
**(A)** Two 3 GHz sinusoidal inputs at −6 dBm with an ITD of *k* cycles between them, and **(B)** the simulated ITD at the injection-locked frequency divider (ILFD) outputs for stage *n*_*best*_ = 23. Left column: simulation results showing the time-averaged outputs of the cochlear cross-correlogram network (using the un-encoded cochlear voltages *V*_*out*_(*n*)) for ITDs of **(C)** *k* = 0, **(E)** $k=\frac{1}{4}$, and **(G)** $k=\frac{1}{2}$ cycles. Right column: similar cross-correlogram results using the frequency- and phase-encoded ILFD outputs for the same ITDs, namely **(D)** *k* = 0, **(F)** $k=\frac{1}{4}$, and **(H)** $k=\frac{1}{2}$ cycles. Comparison of the normalized diagonal lines for different ITDs using **(I)** the un-encoded cochlear outputs (left column) and **(J)** the frequency- and phase-encoded outputs (right column).

As shown in Figure 3, a 2D network of multipliers is used to generate the cross-correlogram matrix **C_AB_**(*t*) between two cochlea chips (denoted cochlea A and cochlea B), each of which has *N* = 50 stages. To illustrate the nature of this matrix, first consider the simplest case of a single CW input source. As an example, we applied 3 GHz sinusoidal inputs at −6 dBm to the two cochleas with an ITD of *k* cycles between them, as shown in Figure 5A. The resulting outputs (from a transistor-level simulation in Cadence Virtuoso) of the ILFDs that are located near the best frequency (*n*_*best*_ = 23 in this case) are shown in Figure 5B. The input ITD is preserved in the phase-locked outputs, as expected.

Figures 5C–H shows the simulated time-averaged cross-correlogram matrices **C_AB,av_** for this CW input at different ITD values. Plots in Figures 5C,E,G correspond to finding the product of the un-encoded cochlear output voltages *V*_*out*_(*n*), while those in Figures 5D,F,H correspond to finding the product of the frequency- and phase-encoded outputs of the ILFDs.

Figure 5C shows the case when ITD is 0, i.e., the two cochlea chips receive identical inputs. The resultant correlation matrix **C_AB,av_** (using the un-encoded cochlear outputs) is positive and non-zero along the diagonal line, with a peak magnitude around the “best” position (*n*_*best*_ = 23) for this frequency. We also observe off-diagonal peaks that are symmetrically distributed about the diagonal; these reflect the correlation between two propagating waves (on the two cochlear transmission lines) at different spatial shifts. Also, note that both diagonal and off-diagonal terms become very close to zero for *n*_*A,B*_>30; this is due to the low-pass nature of the cochlear transfer functions. Figures 5E,G show **C_AB,av_** for non-zero ITDs of $k=\frac{1}{4}$ and $\frac{1}{2}$ cycles, respectively^2^. Clearly, non-zero ITD results in patterns that are asymmetric with respect to the diagonal. For positive ITD, the peak along the diagonal is shifted downward (toward cochlea A). Figure 5I compares normalized values of **C_AB,av_** along the diagonal for different ITDs. These values vary periodically between [−1, 1] as the delay increases (with a period of *k* = 1 cycle), as expected for a cross-correlogram. Thus, the ambiguity-free range for time delay estimation is again [0, π/ω_*i*_] (i.e., *k* = 1/2 RF cycles).

Next, we consider calculating cross-correlograms using the frequency- and phase-encoded outputs. In this case the ILFDs only keep track of the input phase when the input power level exceeds the sensitivity curve for each stage (i.e., that ILFD is locked). Otherwise, the ILFDs are free-running and their time-averaged cross-correlogram tends to zero. As a result, this matrix (denoted by $C_{AB,av}^{Locked}$) is only non-zero over a small region around the best frequency where the ILFDs are locked; in other words, the time delay information becomes highly localized. This property is visible in Figures 5D,F,H, which show $C_{AB,av}^{Locked}$ with the same ITDs as before (0, $\frac{1}{4}$, and $\frac{1}{2}$ cycles). Also note that since the ILFDs have wide locking range, the effects of mismatches between the two cochleas will be removed from $C_{AB,av}^{Locked}$ as long as they remain locked to the input signals. And the phase shift between stages reaches its peak value (70° ~ 80° over 1 GHz ~ 9 GHz) near the CF of each transfer function. These phase shift curves have very similar shapes for all 50 cochlear stages, with some small deviations from scale-invariant behavior. These deviations result in minor differences (<2%) between the peaks of the resulting cross-correlation curves, which can be removed by post-calibration. Also note that since the ILFDs have wide locking range, the effects of mismatches between the two cochleas will be removed from $C_{AB,av}^{Locked}$ as long as they remain locked to the input signals.

Figure 5J compares values of $C_{AB,av}^{Locked}$ along the diagonal for different ITDs. Since the ILFD outputs only encode phase information, all the locked stages have similar cross-correlogram values, as visible in the figure. Interestingly, the ambiguity-free range for ITD estimation in this case is [0, 3π/ω_*i*_] (i.e., *k* = 3/2 RF cycles), which is 3 × larger than before. This is because the phase of each locked ILFD encodes the onset time of its input (see Figures 5A,B for an example). The maximum ITD that can be encoded in this way is 1/2 *output* cycles, which corresponds to 3/2 input (i.e., RF) cycles for a divide-by-3 ILFD. Using a divide-by-*M* ILFD allows this range to be further extended to *M*/2 RF cycles, but at the cost of a more complicated circuit and lower output bandwidth.

### 2.5. Tracking of Moving Sources

One of the key advantages of the proposed source localization method is real-time computation. Since the matrices **C_AB,av_** and $C_{AB,av}^{Locked}$ can be efficiently computed in parallel using the sterausis network, basic information for each source (frequency and ITD) can be estimated within a few ILFD output cycles. As a result, we can track objects that are moving rapidly in (frequency, AOA) space. The ITD Δ*T* (and resulting phase shift Δψ) for an AOA of ϕ is given by

(4) $$\begin{array}{r} \Delta \text{Ψ}=\omega \Delta T=\omega \left(\frac{d}{c}\right)\sin\left(\phi \right), \end{array}$$

where *d* is the distance between the antennas and *c* is the velocity of light. Thus, the rate of change of ITD for a moving source (range *r*, transverse velocity *v*) is given by

(5) $$\begin{array}{r} \frac{d\left(\Delta \psi \right)}{dt}=-\omega \left(\frac{d}{r}\right)\left(\frac{v}{c}\right)\cos\left(\phi \right). \end{array}$$

**Figure 6:**
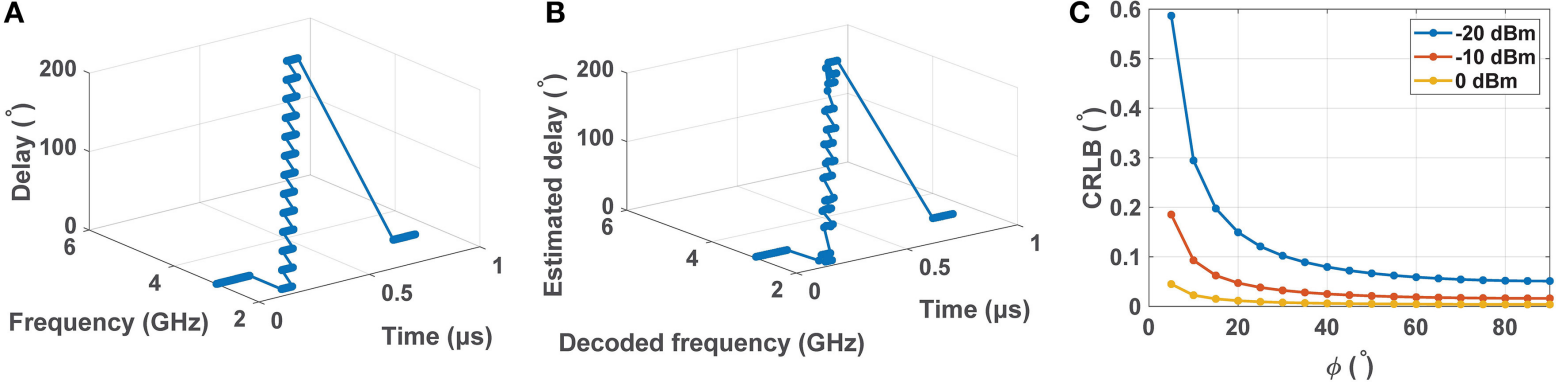
Simulation showing tracking of a rapidly moving source with frequencies and ITDs increasing from 2 to 5 GHz and 0 to π rad, respectively: **(A)** actual trajectory, and **(B)** estimated trajectory for SNR = 20 dB. **(C)** Calculated Cramer–Rao lower bound (CRLB) for the angle of arrival (AOA) estimation (in degrees) at 3 GHz as a function of source position ϕ for various input power levels.

For example, Figure 6A shows a rapidly moving source, whose frequency increases from 2 to 5 GHz as ITD (which encodes AOA) varies from 0 to π rad. In particular, every 50 ns the frequency and ITD increase by 0.25 GHz and 15°, respectively. From Equation (5), these values correspond to *v*≈8.3 × 10^5^ m/s for a 3 GHz source at *r* = 1 m and ϕ = 0, given *d* = 10 cm. The estimated frequencies and ITDs using $C_{AB,av}^{Locked}$ (for an estimation rate = 10 ns/frame and input SNR = 20 dB) are plotted in Figure 6B. The actual and estimated source trajectories are in good agreement, confirming that even such rapidly moving sources can be readily tracked by the network.

### 2.6. Theoretical Bounds on Time Delay Estimation

The accuracy of time delay estimation using the proposed approach is limited by the relevant Cramer-Rao lower bound (CRLB). The latter defines the minimum variance of the estimated time delay as a function of the signal and noise power spectra, and is given by Carter (1987):

(6) $$\begin{array}{r} \sigma^{2}\geq {\left(2T\overset{B}{\underset{0}{\int }}{\left(2\pi f\right)}^{2}\left(\frac{SNR{\left(f\right)}^{2}}{1+2\cdot SNR\left(f\right)}\right)\text{d}f\right)}^{-1}, \end{array}$$

where *T* is the integration time, *B* is the signal bandwidth, and *SNR*(*f*) is the (possibly frequency-dependent) signal-to-noise ratio. Using Equation (4), the time variance σ^2^ can also be transformed into best-case AOA estimation error around a given source position ϕ (Julian et al., 2004):

(7) $$\begin{array}{r} \sigma_{\phi ,min}=\frac{\sigma_{min}}{\Delta T_{max}\sin\left(\phi \right)}, \end{array}$$

where Δ*T*_*max*_≡*d*/*c*. For convenience, we denote σ_ϕ, *min*_ as the CRLB for AOA estimation.

The calculated CRLB for a single source (at 3 GHz) as a function of position ϕ and various input power levels is shown in Figure 6C. Here *SNR*(*f*) was estimated from transistor-level simulations, we have assumed that *T* = 1 ns and *B* = 10 GHz, and the outputs were measured at stage *n* = 25. It is observed that CRLB decreases as the input power level increases, as expected. It is possible to achieve <0.6° estimation error in the range ϕ=[5°, 90°] for a −20 dBm input. Restricting the range to ϕ=[45°, 90°] further reduces the error to <0.1°.

### 2.7. Off-Diagonal Information in the Cross-Correlogram

The limited ambiguity-free range for delay estimation using **C_AB,av_** constrains the antenna separation *d* to small values. In particular, we need *d* ≤ λ_*min*_/4 to obtain full AOA coverage [−π/2,π/2], where λ_*min*_ = *c*/*f*_*max*_ is the wavelength of the maximum input frequency *f*_*max*_. However, such small separation degrades AOA estimation error, since Equation (7) shows that the CRLB σ_ϕ, *min*_∝1/*d*. Thus, there is a fundamental trade-off between ambiguity-free range and accuracy.

The standard approach to relaxing this trade-off is to compute additional products *w*_*A,i*_(*t*)*w*_*B, i*_(*t*+τ) for each element on the diagonal of the cross-correlogram, where τ is a variable time delay parameter (known as the lag). The full cross-correlation vector *x*_*AB, i, i*_(τ) is approximated by time-averaging *K* such product terms, and finally ITD is estimated as the lag where *x*_*AB, i, i*_(τ) reaches its maximum. Unfortunately, this computation is expensive in terms of hardware: each element in **C_AB,av_** now requires its own delay line and a set of *K*≫1 multipliers.

Fortunately, the fact that each cochlea behaves as a transmission line allows the sterausis network to directly compute approximations to *x*_*AB, i, i*_(τ), i.e., eliminates the need for additional area-intensive delay lines (in fact, this was the original motivation for the stereausis algorithm) (Shamma et al., 1989). The process can be explained as follows. Finite wave velocity on the cochlear transmission line results in a group delay τ_*g*_ of several RF cycles before a wave reaches its peak *V*_*out,max*_, which occurs at the “best” stage *n*_*best*_. As a result, cochlear stages before the peak (i.e., *n* < *n*_*best*_) contain approximate copies of *V*_*out,max*_, but with smaller time delays. Multiplying these copies with *V*_*out,max*_ for the other cochlea yields an approximation to *x*_*AB*_(τ). Thus, cross-correlation vectors can be estimated from the off-diagonal components of **C_AB,av_**.

**Figure 7:**
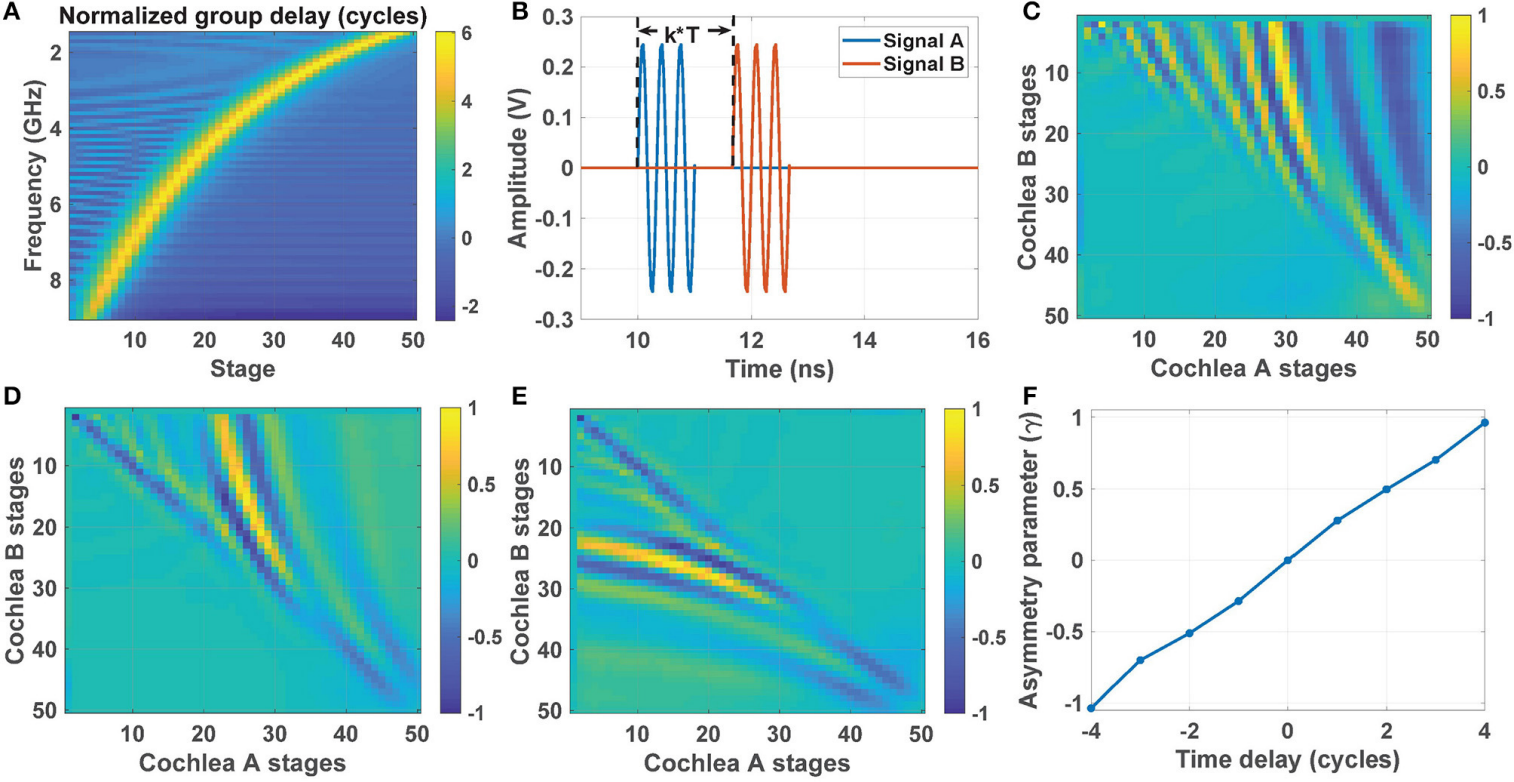
**(A)** Normalized group delay (in cycles) of our cochlear model as a function of input frequency and stage number *n*. **(B)** Pulsed input signals A and B at 3 GHz with an ITD of *k* cycles; here *T* is the RF period. The corresponding cross-correlogram matrices for **(C)** *k* = 5, **(D)** *k* = 3, and **(E)** *k* = −3. **(F)** Estimated asymmetry parameter γ as a function of the time delay (i.e., ITD) in cycles.

The group delay for all 50 stages in our cochlear model (normalized to cycles of the input frequency) is shown in Figure 7A. The maximum group delay occurs near the characteristic frequency ω_*c*_(*n*) of each stage. An average of τ_*g*_≈5.5 cycles is observed for all stages around their characteristic frequencies. To study how this delay is exploited by the stereausis network, consider pulsed input signals A and B at 3 GHz with an ITD of *k* cycles (as shown in Figure 7B). The corresponding cross-correlogram **C_AB,av_** with *k* = 5 is shown in Figure 7C. The peaks are shifted away from the diagonal and toward cochlea A, and maximized around stage *n*_*best*_ = 30. The shift decreases when ITD is reduced to *k* = 3 (Figure 7D), and the maximum also moves to *n*_*best*_ = 25. The resulting pattern is reflected about the diagonal when the ITD becomes negative. Thus, the peaks in Figure 7E (*k* = −3) are shifted toward cochlea B.

Thus, ITD over a broad range can be efficiently calculated by quantifying the asymmetry of the cross-correlogram matrix **C_AB,av_** with respect to the main diagonal (Julian et al., 2004). A suitable asymmetry parameter γ can be defined as the weighted difference between upper and lower triangular elements:

(8) $$\gamma =\sum_{i<j}(j-i)\times c_{AB,i,j}^{2}-\sum_{i>j}\left(i-j\right)\times c_{AB,i,j}^{2}.$$

Figure 7E shows the calculated value of γ (after normalization to the [−1,1] range) for the pulsed inputs shown in Figure 7B. The value of γ is approximately linear with ITD over the range [−4, 4] cycles, thus significantly relaxing the range-accuracy trade-off for AOA estimation.

### 2.8. Localization in Complex Environments

Binaural processing is known to improve sound perception in complex acoustic environments (Mead et al., 1991). Unlike earlier correlation-based binaural localization algorithms, which generally localize only the strongest source at any given time, the proposed cochlea-based system has *N* = 50 parallel output channels that can be used to simultaneously localize multiple sources. However, these sources have to be far enough apart in the frequency domain to generate distinct peaks and/or phase-locked regions in the cochlear outputs. Note that $C_{AB,av}^{Locked}$ has sharp and well-separated peaks due to the on/off nature of phase-locking. The widths of the peaks (i.e., the phase-locked regions) do increase with input power level, which limits the ability to localize multiple weak sources in the presence of strong blockers. However, the peaks have very similar amplitudes because all the ILFDs generate logic-level signals. On the other hand, the cochlear transfer functions are broader and have different peak gains (as shown in Figure 4B), which result in different peak amplitudes within **C_AB,av_**. Thus, ILFD-based cross-correlograms are more suitable for analyzing multi-source environments.

**Figure 8:**
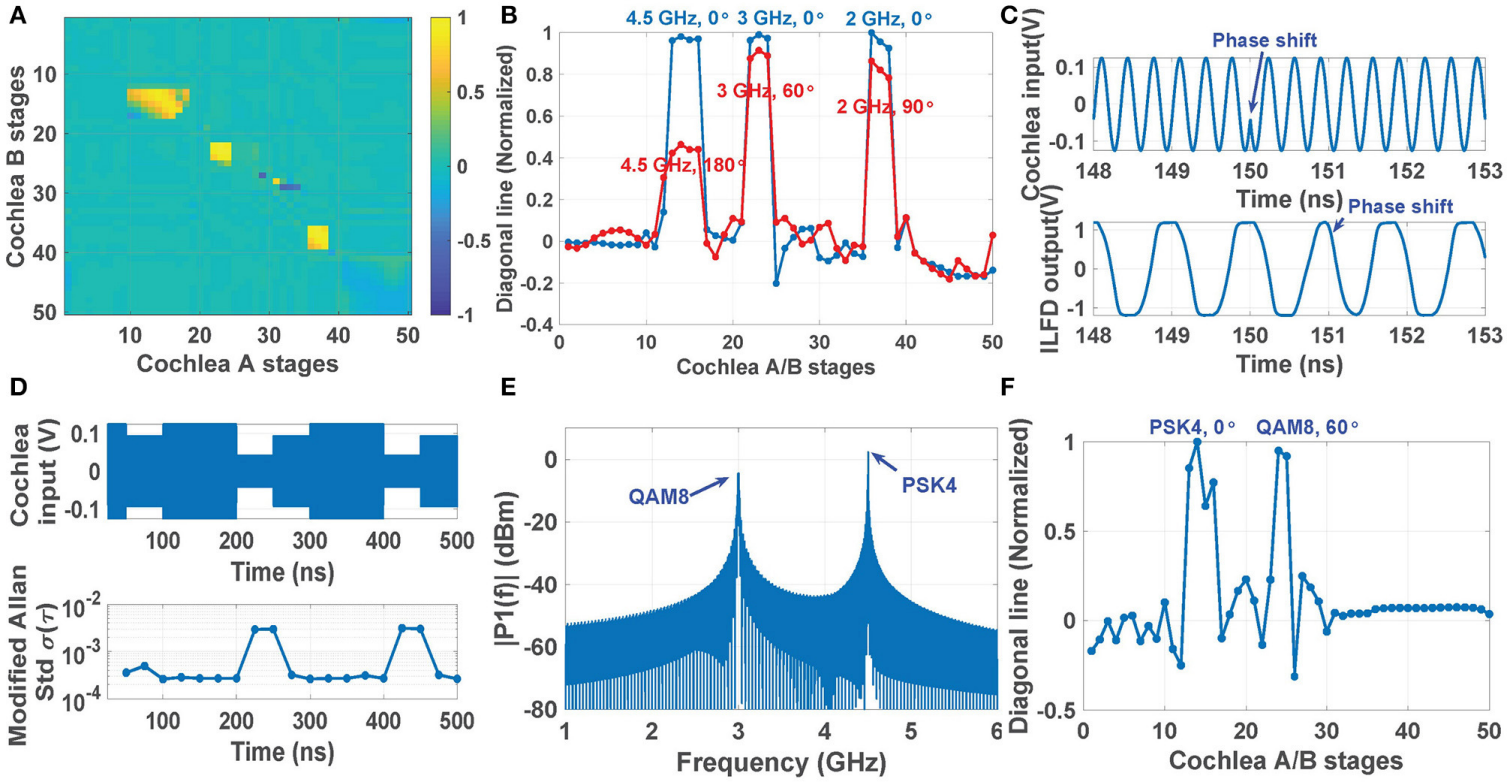
Simulation results showing the ILFD-based cross-correlogram generated by a three-tone input: **(A)** 4.5, 3, and 2 GHz with all ITDs equal to 0; **(B)** the corresponding normalized diagonal line (blue line), compared with the diagonal line with non-zero ITDs, namely [π,π/3,π/2] (red line). **(C)** Time domain waveforms of a QAM8-modulated cochlea input and the resulting ILFD output during a symbol transition. **(D)** Modified Allen deviation of a single stage output (for averaging time τ = 5.12 ns) estimated each 25 ns when the cochlea receives a QAM16 input with a symbol rate of 20 MS/s; **(E)** Measured power spectrum of an input signal consisting of two modulation types (PSK4 and QAM8), and **(F)** its ILFD-based cross-correlogram.

Figure 8A shows the phase-encoded cross-correlogram $C_{AB,av}^{Locked}$ in response to the simultaneous presentation of 2, 3, and 4.5 GHz CW sources, all with an ITD of 0. The resulting patterns from each source are well-separated and thus can be easily localized in parallel. The blue curve in Figure 8B shows the values along the diagonal for the same input. Three peaks are clearly visible: stages 13~16, 22~24, and 36~38 are phase-locked to the sources at 4.5, 3, and 2 GHz, respectively. The ITD vector for these sources is now changed to a non-trivial value, namely [π,π/3,π/2], and the resulting diagonal elements in $C_{AB,av}^{Locked}$ are shown as the blue curve in Figure 8B. Finally, the amplitudes of the three peaks are used to estimate AOA; the result is ϕ=[185.4°, 67.6°, 90.7°], which has an average error of 4.9%.

Since the ILFDs (which are analogous to phase-locked auditory neurons) have wide locking and tracking bandwidths, the proposed cross-correlogram based source localization approach is not restricted to narrowband CW inputs, and can readily handle typical digitally-modulated wireless signals. To demonstrate this point, MATLAB was used to generate the frequency-time spectra of wideband signals based on one of several common digital modulation schemes (e.g., QAM8, QAM16, PSK4, etc.), and the parallel ILFD outputs kept under observation. When a phase shift occurs in the cochlear input (e.g., due to a symbol transition), the output of a locked ILFD will lock to the new input phase within a couple of cycles (as shown in Figure 8C for the case of QAM8). However, when the new symbol has low enough input amplitude, the ILFD can become transiently unlocked. In this case the modified Allen deviation σ_*A*_(τ) of the locked outputs (which can be easily measured using a frequency counter) increases significantly as shown in Figure 8D for the case of QAM16. In this case, the figure shows that σ_*A*_(τ) increases by approximately 10 × between the locked and unlocked states (from ~4 × 10^−3^ to ~4 × 10^−2^, respectively). Thus, greater source localization accuracy can be ensured by monitoring σ_*A*_(τ) and comparing it with a threshold to reject such unlocked intervals before the cross-correlogram is computed.

It is also possible to simultaneously localize multiple modulated sources. For example, Figure 8E considers the case when the scene contains two spatially-separated modulated sources (in this case, PSK4 at 4.5 GHz with a phase shift of 0 rad, and QAM8 at 3.0 GHz with a phase shift of π/3 rad). The resulting diagonal elements of the ILFD-based cross-correlogram are shown in Figure 8F. The two modulated signals are locked over different cochlear stages (stages 13~16 and 24~25, respectively), thus allowing the two sources to be independently demodulated and localized.

## 3. Experimental Results

### 3.1. Performance of the RF Cochlea Chip

**Figure 9:**
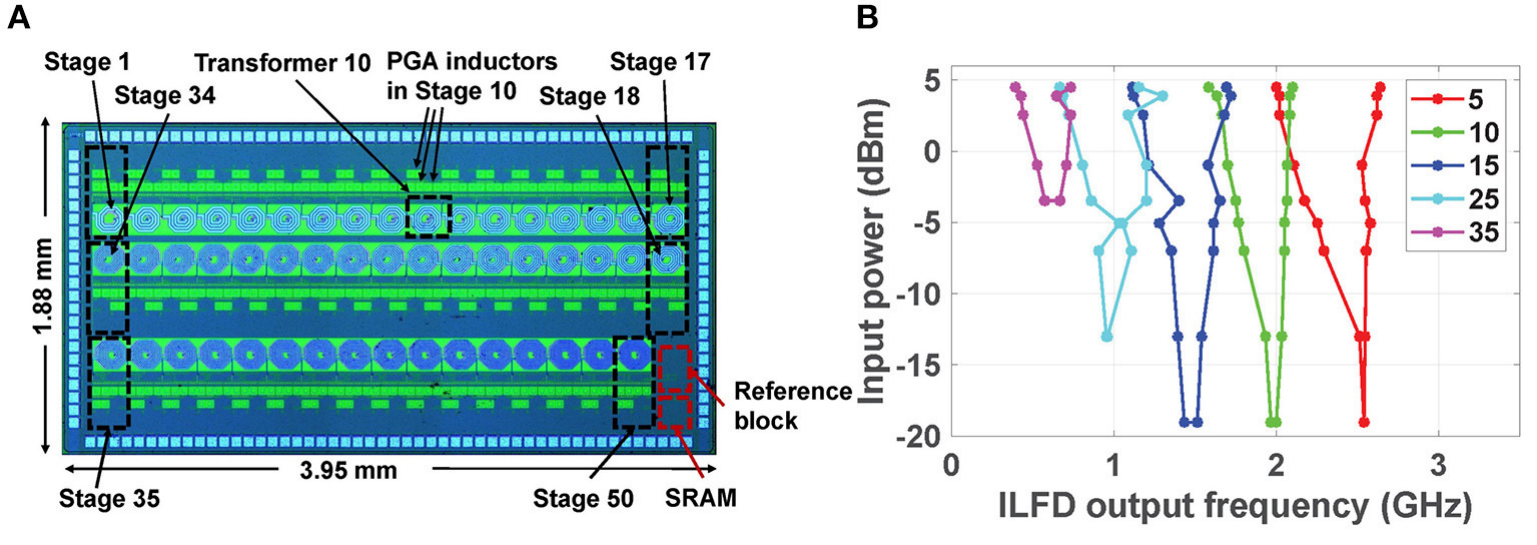
**(A)** Die photograph of the digitally-programmable RF cochlea chip; and **(B)** measured input sensitivity curves for the frequency-encoded outputs at stages {5, 10, 15, 25, 35} (Wang et al., 2020).

The RF cochlea chip was fabricated in 65 nm CMOS, as shown in Figure 9A. This chip consumes 418 mW and typically generates ~1 GS/s of total data at an ENOB of 5-6 bits. The frequency encoder in each stage uses a ring-oscillator-based divide-by-3 ILFD for locking to the input signal after cochlear filtering, followed by several stages of static frequency division implemented using current-mode logic (CML) latches. Figure 9B shows the measured input sensitivity curves of the ILFDs (known as Arnold tongues) over a broad set of stages, namely {5, 10, 15, 25, 35}. The Arnold tongues shift to lower frequencies as we move toward the apex (i.e., the stage number increases), similar to the mammalian cochlea. Table 1 summarizes the measured performance of this design (Wang et al., 2020).

**Table 1:** Cochlea chip performance summary.

**Parameter**	**Value**
CMOS technology	UMC 65 nm
Frequency range	1.0–8.3 GHz
Peak voltage gain	12 dB
Power consumption	418 mW
Dynamic range	62 dB
Area	3.95 × 1.88 mm
Output	50 LVDS pairs

In addition, simulations of an on-chip XOR-based stereausis network show a power consumption of 715 mW (for an output update rate of 20 MHz) and a layout area of ~2.875 mm^2^. Thus, the stereausis network only modestly increases the power and area requirements of the system (relative to the two cochleas alone)—by 85 and 20%, respectively.

### 3.2. Test Setup

To analyze the phase information present at the outputs of the frequency encoders in the selected cochlear channels, we assume single-tone CW input signals for channel A and channel B are sin(2π*ft*) and sin(2π*ft*−ψ_*d*_), respectively, where ψ_*d*_ = 2π*fτ*_*d*_ is the phase difference between the two channels for an ITD of τ_*d*_. The phase difference between the ILFD outputs is then $\frac{\psi_{d}}{3}$, and it is further decreased by a cascade of D-type frequency dividers (FDs) and becomes $\frac{\psi_{d}}{3\times 2^{M}}$ at the cochlea output, where *M* is the number of FD stages.

Since the ILFD and FD outputs are logic-level signals (square waves), their phase shifts can be efficiently estimated using on-chip XOR gates, as mentioned earlier. However, our experimental prototype used off-chip components. Since discrete logic gates do not have enough bandwidth, we replaced them with high-speed four-quadrant analog multipliers (AD834, Analog Devices). These devices have a bandwidth of 500 MHz, which is sufficient for processing the fundamental component of the FD outputs (but not their harmonics). Thus, their outputs are given by

(9) $$\begin{array}{r} \begin{array}{c} c_{AB}\left(t\right)=\sin\left(2\pi \frac{f}{3\times 2^{M}}t\right)\times \sin\left(2\pi \frac{f}{3\times 2^{M}}t-\frac{\psi_{d}}{3\times 2^{M}}\right),\\ =\frac{1}{2}\left(\cos\left(\frac{\psi_{d}}{3\times 2^{M}}\right)-\cos\left(\frac{\pi f}{3\times 2^{M-2}}t\right)\right). \end{array} \end{array}$$

After low-pass filtering, the cross-correlogram output is

(10) $$\begin{array}{r} c_{AB,av}=\frac{1}{2}\cos\left(\frac{\psi_{d}}{3\times 2^{M}}\right)=\frac{1}{2}\cos\left(\frac{\pi f}{3\times 2^{M-1}}\times \tau_{d}\right). \end{array}$$

Thus, the output voltage of the multiplier allows estimation of the input ITD τ_*d*_, and hence the AOA of the transmitter.

**Figure 10:**
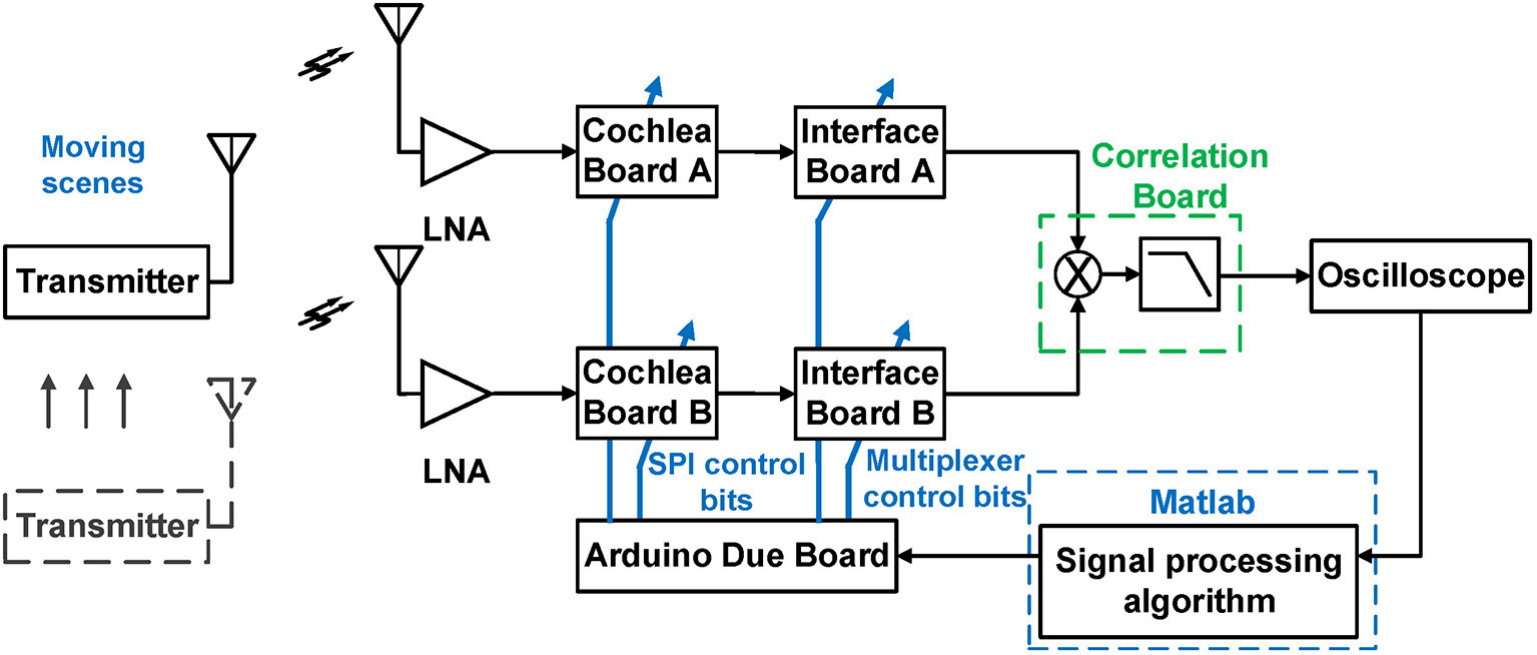
Block diagram of an experimental prototype of a two-channel RF scene analysis system based on two RF cochlea chips.

Figure 10 shows our experimental prototype, which used two RF cochlea test boards and a set of analog multipliers integrated on another board. Signals are fed into the cochleas through two broadband RF front-ends, each of which includes a Vivaldi antenna and an off-the-shelf low-noise amplifier (LNA).

The *N* = 50 parallel outputs from the two cochleas are multiplexed through an interface board (to reduce the number of multipliers in this initial implementation) and fed into the custom cross-correlogram board. The latter contains four parallel channels, each consisting of a four-quadrant multiplier (AD834) and an op-amp based active low-pass filter. The output voltages of these channels are digitized (in this prototype, by a digital oscilloscope) and processed in MATLAB to estimate AOA values for the sources detected by the four selected cochlear channels. These selections can be changed by the MATLAB algorithm (e.g., to adapt to a change in source frequency) by programming a microcontroller (Arduino Due) which in turn programs the multiplexer on the interface board. In addition, the microcontroller can program the gains of the cochlear stages via the chip's built-in SPI port; this is useful for automatic gain control (AGC) to increase the system's DR.

### 3.3. Over-the-Air RF Source Localization

**Figure 11:**
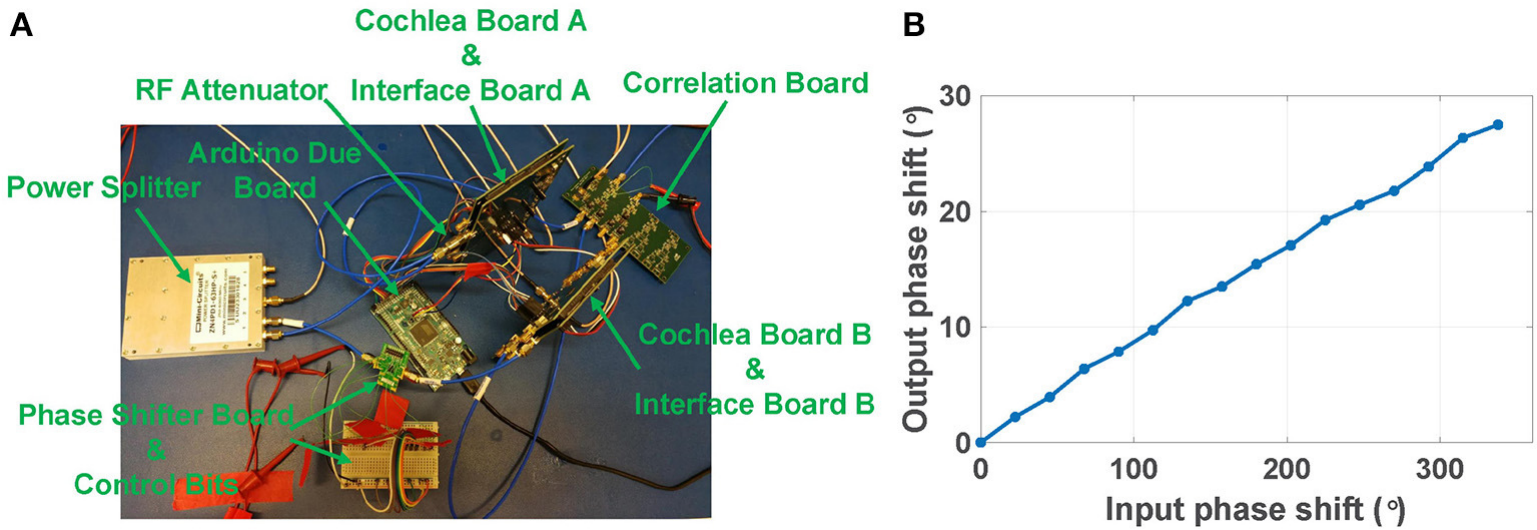
**(A)** Experimental setup of a two-channel RF scene analysis system with ideal input signals. **(B)** Calculated phase shift vs. actual input phase shift for the two RF cochlea boards (channels A and B).

In the first experiment, we fed signals from a RF signal generator directly to the inputs of the two RF cochleas. As shown in Figure 11A, both channels are fed the same signal from a RF power splitter, except for a time delay applied to the signal in channel B (relative to that in channel A) using a digitally-programmable RF phase shifter (HMC649ALP6E, Analog Devices). In addition, a 6 dB RF attenuator at the input of channel A is used to compensate the insertion loss of the phase shifter. The input power level to the cochleas was kept high enough (~0 dBm) to ensure phase-locked ILFD outputs.

The phase shifter has 6-bit control over the range [0°, 360°], resulting in an LSB = 5.625°. We kept the input frequency fixed at 3.4 GHz, swept the phase shift, and extracted the resulting ITD by averaging the output of the analog multiplier over each phase shift step. Figure 11B shows the calculated output phase shift Δψ_*out*_ as a function of the actual input phase shift Δψ_*in*_. As Δψ_*in*_ increases from 0° to 360°, the estimated value of Δψ_*out*_ linearly increases from 0° to 30° as expected; this is because the total frequency division ratio was set to $\frac{1}{3\times 2^{M}}$ with *M* = 2, i.e., a value of 1/12. The input signal amplitude at the multiplier board for $\Delta \psi_{in}=0^{{}^{\circ}}$ was used to calibrate the estimate for Δψ_*out*_.

**Figure 12:**
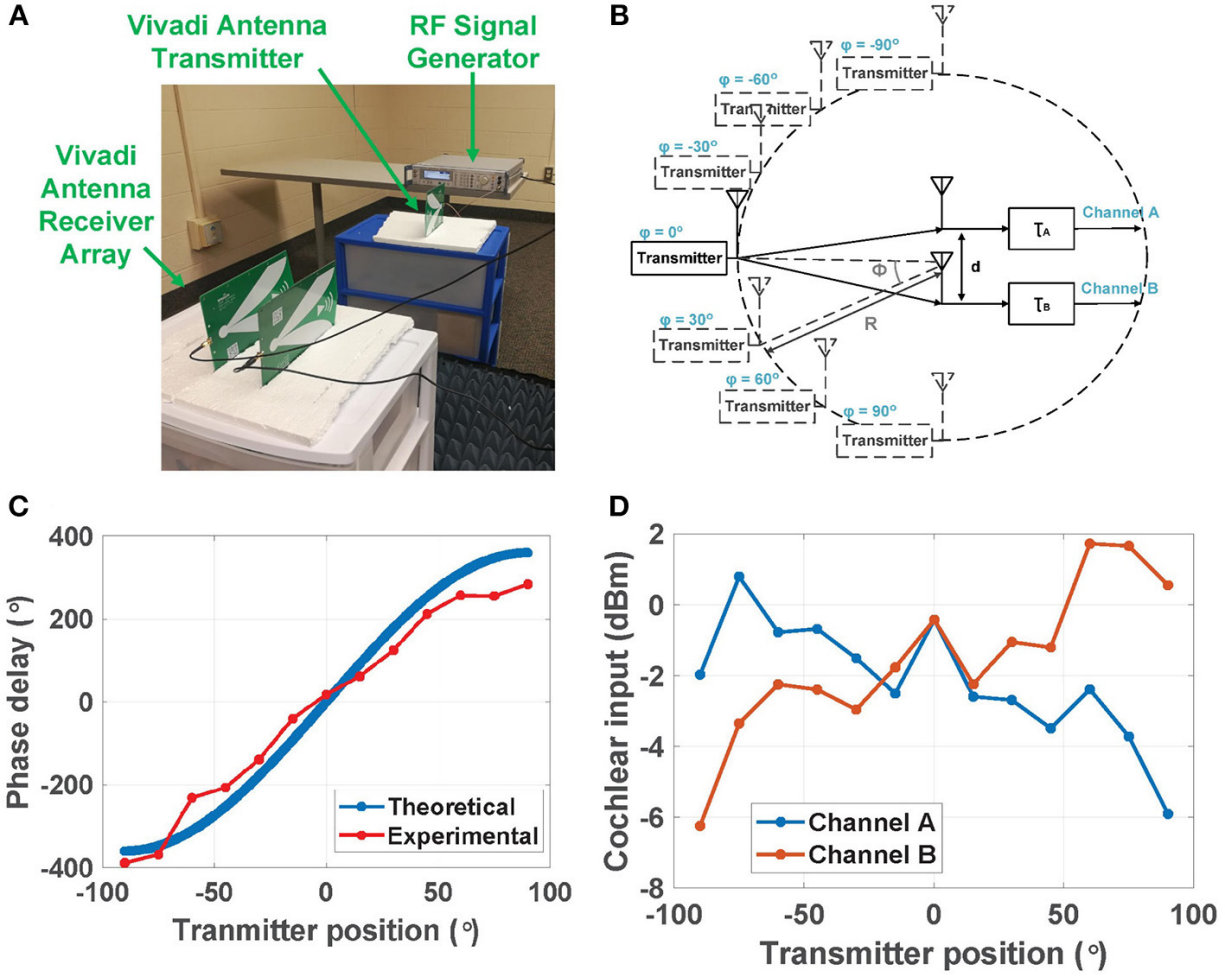
**(A)** Experimental setup of a two-channel RF scene analysis system used for over-the-air localization experiments at 3.0 GHz. **(B)** Positions of the transmitter (moving around the circumference) and the two receive antennas (kept fixed). **(C)** Theoretical and experimental phase delay Δψ (i.e., ITD) between the two channels at different transmitter positions; and **(D)** experimental input power levels for the two channels at different transmitter positions.

In the second experiment, we attempted to extract ITD in a more realistic over-the-air indoor environment with RF signals received by the two planar Vivaldi antennas. Figure 12A shows the experimental setup and Figure 12B shows a simplified block diagram of the positions of the transmitter and the two receiver antennas. The two receivers were placed symmetrically (with spacing *d*) about the center of a circle with radius *R*≈1 m, which is large enough to ensure far-field conditions at the receivers. The transmitter was moved around the circumference of this circle for azimuthal angles in the range ϕ= [−90°, 90°]. We denote the propagation delays to the two receivers by τ_*A*_ and τ_*B*_. To ensure that |τ_*A*_−τ_*B*_| ≤ *T* (i.e., one RF period) such that the ITD is resolvable without ambiguity, we kept the RF frequency fixed at *f*_*in*_ = 3.0 GHz and set *d* = λ = 10 cm. The results can be easily generalized to other input frequencies and also multiple sources, as discussed in section 2.

Figure 12C compares the theoretical and experimental ITD Δψ between the two channels for transmitter positions in the range ϕ= [−90°, 90°] and a step size of Δϕ = 15°. The theoretical ITD was calculated using Equation (4). The experimental ITD was calibrated using the measured output voltage at Δψ_*in*_ = 0 in the first experiment (i.e., using wired inputs). The theoretical and measured ITD curves are in good agreement.

Figure 12D also shows the estimated input-referred power levels *P*_*in*_ for the two cochlear channels vs. transmitter position ϕ. These values were estimated by i) converting the digitized outputs of the amplitude encoding circuits to output voltages *V*_*out*_; and ii) finding *P*_*in*_ by dividing *P*_*out*_ with the cochlear transfer function. It is difficult to calculate theoretical values for *P*_*in,A*_ and *P*_*in,B*_ due to uncertainties in the antenna gains and wireless path losses. However, the experimental values reveal an approximately linear dependence between *P*_*in*_ (in dB) and ϕ, with opposite slopes for the two antennas. This dependence is due to *physical self-shadowing*: one receive antenna blocks (i.e., shadows) part of the wave incident on the other antenna. This effect increases with ϕ because of the planar antenna geometry (see Figure 12A). Finally, ILD can be estimated as the ratio of input power levels, i.e., ILD ≡*P*_*in,A*_/*P*_*in,B*_.

**Figure 13:**
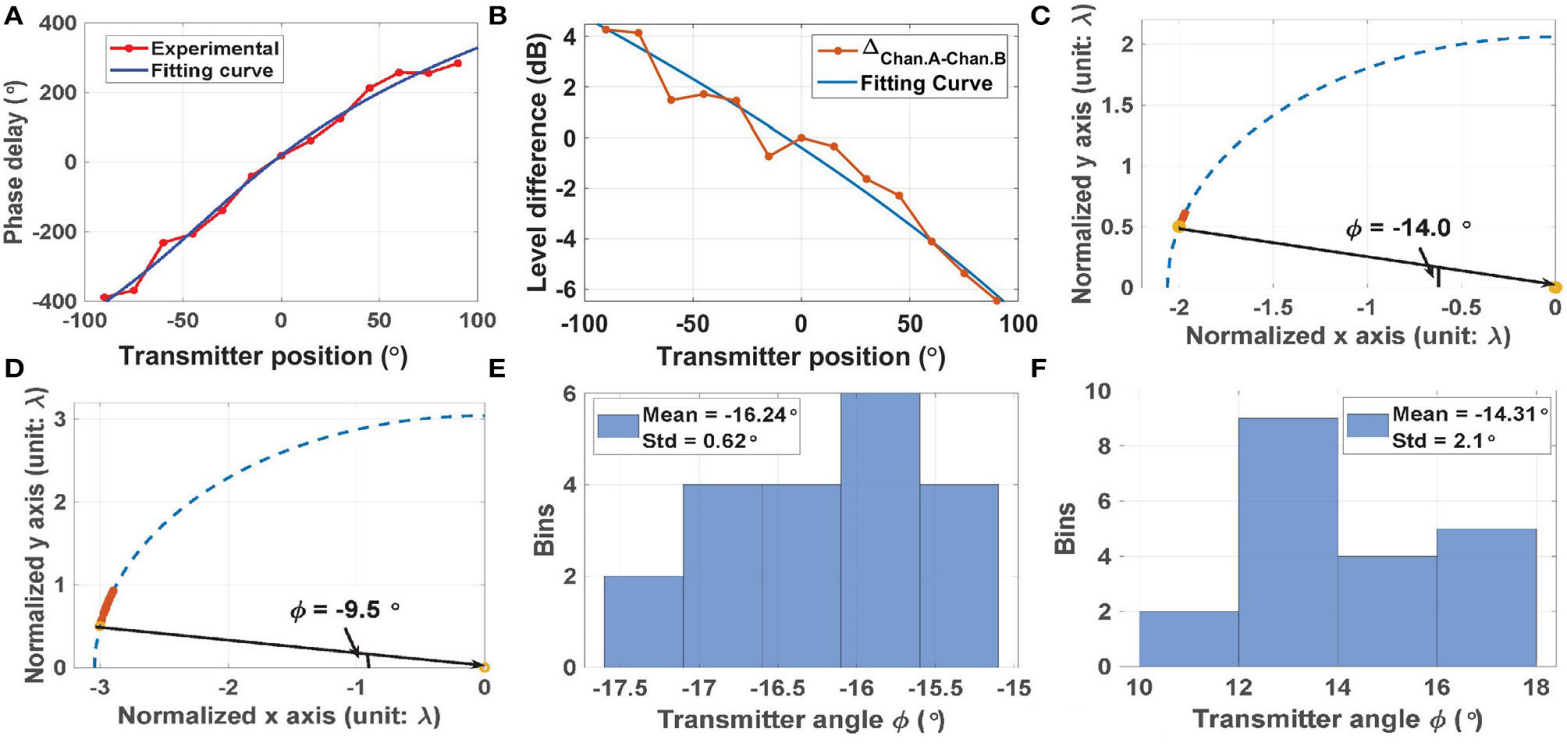
**(A)** Experimental cochlear phase delay (i.e., ITD) and a polynomial fitting curve. **(B)** Experimental cochlear input power differences (i.e., ILD) and a polynomial fitting curve. Estimated transmitter positions using **(C)** ITD, and **(D)** ILD. In both cases, results from *N* = 20 trials are shown, and the actual transmitter position is indicated by the yellow circle. **(E.F)** Histograms of the position estimation error using **(E)** ITD, and **(D)** ILD.

In the next experiment, we built and tested data-driven models for localizing the transmitter using (i) ITD, and (ii) ILD. For this purpose, the experimental data in Figures 12A,B was fit to third- and second-order polynomials, respectively, as shown in Figures 13A,B. Over the full range [−90°, 90°], using the ITD method (as shown in Figure 13A) results in a mean fitting error ϵ = 4.7° and a standard deviation σ = 10.7°, while using the ILD method (as shown in Figure 13B) results in a smaller mean fitting error ϵ = 0.9°, but a larger standard deviation σ = 12.4°. These errors are small enough to confirm the utility of our approach in real-life wireless environments.

The fitted curves were used to localize sources at (i) ϕ = −14.0° (using ITD, results shown in Figure 13C); and (ii) ϕ = −9.5° (using ILD, results shown in Figure 13D). In both cases, the red stars denote the estimated source locations (*N* = 20 trials), while the yellow circle shows the actual location (i.e., the ground truth). The histograms of estimation error in both cases are plotted in Figures 13E,F. These results show that ITD is more accurate than ILD in this case; it provides ~3 × smaller values of mean error and standard deviation (ϵ = 2.2° and σ = 0.62°, respectively). In addition, the ITD results are robust to parameter mismatch between the cochleas as long as the ILFDs remain locked, while the accuracy of the ILD results degrade with mismatch. Fortunately, Monte-Carlo circuit simulations show that the magnitudes of the cochlear transfer functions are tolerant to process corners and device mismatch (standard deviation σ <5%). The main reason for such robustness is the extensive use of well-matched passive on-chip inductors and capacitors to define the transfer functions, rather than the active circuits used to realize audio-frequency silicon cochleas.

The human auditory system mainly relies on ITDs for localization at frequencies <1 kHz (where neural phase locking occurs but self-shadowing effects are small), and mainly on IIDs at frequencies >1.5 kHz (where phase locking is ineffective but self-shadowing by the head becomes significant). There is also a transition zone between 1 and 1.5 kHz where both mechanisms play a role. Given similar wave physics, we expect ITD- and ILD-based source localization methods to also have complementary advantages at RF. It is therefore of interest to combine them to create a better localization model. The two most common range-based non-GPS localization methods, i.e., RSSI and AOA, have been intensively studied at RF over the last decade. However, an in-depth analysis of methods to combine these two cues is still unavailable, although weighted sums have been proposed (Nguyen et al., 2019). We will study such combinations in our future work.

### 3.4. Range Dependence of Over-the-Air Source Localization

Let us define *R* as the radial distance (i.e., range) of an RF source from the origin (see Figure 2A). The received power from this source decreases as 1/*R*^2^ in free space, and approximately as 1/*R*^*n*^ in scattering environments (where *n* = 2–4). The minimum useful source range *R*_*min*_ for the proposed localization method is limited by saturation of the cochlea (which occurs for input power levels >5 dBm), while the maximum useful range *R*_*max*_ is limited by either the ILFD locking threshold (for ITD) or circuit noise (for ILD). For example, as source distance increases and the input power level drops, it eventually falls below the relevant ILFD's locking threshold. As a result, the ILFD becomes unlocked and its average cross-correlation $C_{AB,av}^{Locked}$ becomes zero, thus preventing ITD-based localization. The locking thresholds are frequency-dependent (as shown in Figure 4C) but generally range from −10 dBm to −20 dBm, thus limiting the useful localization range *R*_*max*_/*R*_*min*_ for a given source. Fortunately, the range can be greatly increased by using automatic gain control (AGC). In this approach, a programmable gain amplifier (PGA) is placed after each LNA (see Figure 3), and its gain adjusted to keep the signal power at the cochlear input terminals relatively constant.

Similarly, the maximum localization range *R*_*max*_ using ILDs is limited by the SNR of the cochlear outputs. For our current design, the maximum available SNR is $SNR_{max}\approx \left(V_{L}^{2}/2\right)/\bar{v_{n,out}^{2}}$ where *V*_*L*_ is the linear range of the active circuit within each cochlear stage, and $\bar{v_{n,out}^{2}}$ is the total output noise. Assuming a reasonable value of *V*_*L*_ = 0.2 V, $\bar{v_{n,out}^{2}}$ and *SNR*_*max*_ vary over the ranges 210-330 μV_*rms*_ and 52-57 dB, respectively. Thus, the useful dynamic range (DR) for ILD-based localization is ~50 dB, which corresponds to a *R*_*max*_/*R*_*min*_ ratio of about 300 × in free space. Again, this range can be further extended using an AGC if required.

### 3.5. Comparison to Prior Work

Table 2 summarizes the performance of this design and compares it with prior work on bio-inspired source localization. Ours is the only work that operates at RF (earlier efforts were limited to audio frequencies). Also, our work has the lowest source localization errors for both ITD- and ILD-based methods.

**Table 2:** Performance summary compared with prior work.

**References**	**Cues**	**Stimulus**	**RMS error**	**Approach**
			**(0^**°**^-45^**°**^/45^**°**^-90^**°**^)**	
This work	ITD, ILD	Sine tones	0.6°(ITD),	Two silicon cochleas
		(1.0–8.3 GHz)	2.0°(ILD)	
Xu et al. (2019)	IPD,	Austalk	3.68°	Two digital cochleas
	Spectral cues			(CNN)
Chan et al. (2010)	ITD	Sine tones	2.7°/5.5°	Two silicon cochleas
		(400, 650 Hz)		(AER-EAR)
van Schaik and Shamma (2003)	ITD	Sine tones	3°/12°	Two silicon cochleas
		(50–300 Hz)		(zero-crossing)

## 4. Conclusion

We have described a hardware-efficient, real-time, ultra-wideband, and multi-source RF localization system based on combining two biologically-inspired broadband RF signal analyzers (“RF cochleas”) with a stereausis network that generates cross-correlograms from the cochlear outputs. We have demonstrated the operation of the proposed system using both simulations and preliminary over-the-air wireless tests. Realistic indoor and outdoor wireless channels are subject to additional effects, including wide-band interference and multi-path propagation. The effects of interferers on source localization can be greatly reduced by using a set of tunable band-pass or band-stop filters before the cochleas, as shown in Figure 3. The effects of multi-path propagation on source localization in various RF environments are harder to model and predict, and will be studied in our future work.

Future work will focus on extending the proposed source localization method to the elevation plane. One method is based on monaural cues, for example intensity differences between adjacent output channels of the same cochlea (Searle et al., 1976). These differences arise from the frequency-dependent radiation patterns of the two antennas. Another promising approach is based on modifying the radiation patterns of the two antennas such that they become asymmetric with respect to the azimuth plane (e.g., by rotating one of them); this makes the binaural cues (mainly the ILD) elevation-dependent. Note that the two ears of the barn owl (*Tyto alba*) are asymmetrically positioned within its face to generate similar azimuth- and elevation-dependent binaural cues (Knudsen and Konishi, 1979); the bird uses these cues to accurately localize acoustic sources in 2D.

## Data Availability Statement

The original contributions presented in the study are included in the article/supplementary material, further inquiries can be directed to the corresponding author/s.

## Author Contributions

YW performed the simulations and experiments. SM designed the overall system and carried out the theoretical analysis. Both authors wrote the manuscript.

## Conflict of Interest

The authors declare that the research was conducted in the absence of any commercial or financial relationships that could be construed as a potential conflict of interest.

## Footnotes

^1^The DAS beamformer is an exception; it can be implemented using analog phase shifters or delay lines.

^2^Positive ITD values imply that the inputs to cochlea B are delayed compared to cochlea A.
